# Methods of solving the system of equations for the energy gap in the revisited BCS theory of superconductivity

**DOI:** 10.1016/j.mex.2021.101388

**Published:** 2021-05-19

**Authors:** Dragoş-Victor Anghel, Amanda Teodora Preda

**Affiliations:** a Horia Hulubei National Institute for Physics and Nuclear Engineering, P.O. Box MG-6, 077126 Măgurele, Ilfov, Romania; b University of Bucharest, Faculty of Physics, Romania

**Keywords:** Superconductivity, BCS theory, BCS gap equation, Quantum statistics, Phase transitions

## Abstract

The Bardeen-Cooper-Schrieffer (BCS) theory of superconductivity has been revisited in a series of papers [1], [2], [3] and in this context the equation for the energy gap was generalized to a system of integral equations. The physical consequences of this change are major, leading not only to the change of the critical temperature and of energy gap, but even to a change of the order of the phase transition and to multiple solutions for the energy gap. Nevertheless, finding the solutions of the proposed system of equations is much more complicated than solving the typical BCS gap equation and requires a careful analysis. This analysis is done here and consists of the following steps:

• writing the system of equations at finite temperature ($k_{B}T$ comparable with the energy gap Δ) and in the low temperature limit ($k_{B}T\ll \Delta$);
• separate analysis of the equations and of their solutions in the two temperature ranges, (first) $k_{B}T\ll \Delta$ and (second) $k_{B}T$ comparable with Δ;
• presenting the methods to consistenlty searching the solutions.

## Graphical abstract

**Figure:**
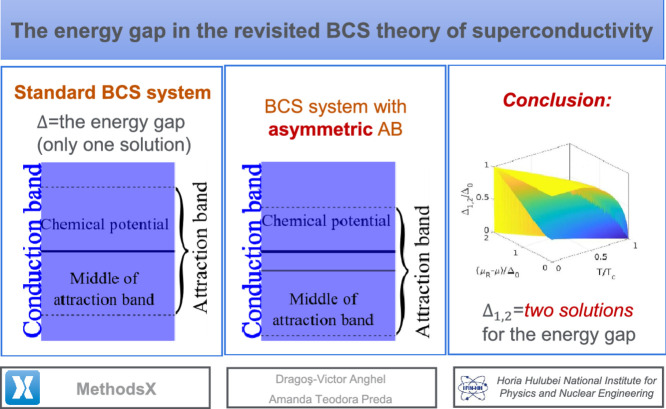

Specifications table

**Table:** 

Subject Area	Physics and Astronomy
More specific subject area	*Condensed Matter Physics*
Method name	*Multiple solutions for the BCS energy gap system of equations*
Name and reference of original method	*BCS energy gap equation*
Resource availability	

## Method details

Let us detail here the methods used in Ref. [3]. The starting point is the BCS Hamiltonian [4]

(1) $$\hat{H}=\sum_{ks}\in_{k}^{\left(0\right)}{\hat{n}}_{ks}+\sum_{kl}V_{kl}c_{k↑}^{†}c_{-k↓}^{†}c_{-l↓}c_{l↑},$$

written in terms of the creation $c_{k,s}^{†}$ and annihilation $c_{k,s}$ operators on the states determined by the quantum numbers (k,s) (concretely, k is the wavevector and s≡↑,↓ is the spin projection). The free particle energy is $\in_{k}^{\left(0\right)}$ and the number operator is denoted by ${\hat{n}}_{ks}\equiv c_{k,s}^{†}c_{k,s}$. We assume $V_{kl}\equiv -V$ if $\in_{k}^{\left(0\right)},\in_{l}^{\left(0\right)}\in I_{V}\equiv \left[\mu -\hbar \omega_{c},\mu +\hbar \omega_{c}\right]$ and $V_{kl}\equiv 0$ otherwise. Then, $\mu \hat{N}$ is subtracted from the Hamiltonian $\left(\hat{N}\equiv \sum_{k,s}c_{\mathrm{ks}}^{†}c_{\mathrm{ks}}\right)$ and ${\hat{H}}_{M}=\hat{H}-\mu \hat{N}$ was liniarized and diagonalized by the Bogoliubov-Valatin transformations [5,6],

(2) $${\hat{H}}_{M}=\sum_{k}\left(\xi_{k}-\in_{k}+\Delta b_{k}^{*}\right)+\sum_{k}\in_{k}\left(\gamma_{k0}^{†}\gamma_{k0}+\gamma_{k1}^{†}\gamma_{k1}\right)$$

where $\xi_{k}\equiv \in_{k}^{\left(0\right)}-\mu$, $\in_{k}\equiv \sqrt{\xi_{k}^{2}+\Delta^{2}}$, Δ is the *energy gap*, $\gamma_{k0}=u_{k}c_{k↑}-v_{k}c_{-k↓}^{†}$, and $\gamma_{k1}=v_{k}c_{k↑}^{†}+u_{k}c_{-k↓}$ ($\gamma^{†}$ and γ are *quasiparticle creation and annihilation operators*, respectively). The coefficients $u_{k}$ and $v_{k}$ satisfy

(3) $$\left|v_{k}{|}^{2}=1-\right|u_{k}{|}^{2}=\frac{1}{2}\left(1-\frac{\xi_{k}}{\in_{k}}\right).$$

In these notations, the energy gap satisfies [4,1],

(4) $$1=\frac{V}{2}\sum_{k}\left(\frac{1-n_{k0}-n_{k1}}{\in_{k}}\right)$$

Eq. (4) ensures the consistency of the formalism.

In equilibrium, the populations $n_{ki}$ are functions of temperature, as we shall see below.

### Standard formalism

In the standard formalism, μ is the chemical potential and the quasiparticle populations are [4]

(5) $$n_{ki}\equiv \langle \gamma_{ki}^{†}\gamma_{ki}\rangle =\frac{1}{e^{\beta \in_{k}}+1},\;i=0,1,$$

where $\beta \equiv 1/(k_{B}T)$ and $k_{B}$ is the Boltzmann's constant. Plugging Eq. (5) into Eq. (4), one obtains an equation in the unknown energy gap Δ. Since the wavevector k influences the populations only through the single-particle energies $\xi_{k}$ and $\in_{k}$, in the following we shall drop the subscript k and we shall replace the summations over k by (formal) summations over ξ or ∈ (preserving the number of states), whenever this does not lead to confusion. Furthermore, since we analyze large (macroscopic) systems, we introduce the density of states $\sigma_{0}$ along the ξ (or $\in^{\left(0\right)}$) axis and replace the summations by integrals. In the case of Eq. (4), this leads to

(6) $$\frac{1}{\sigma_{0}V}=\int_{0}^{\hbar \omega_{c}}\frac{1-2n_{\xi }\left(T\right)}{\in_{\xi }}d\xi =\int_{\Delta }^{\sqrt{{\left(\hbar \omega_{c}\right)}^{2}+\Delta^{2}}}\frac{1-2n_{\in }\left(T\right)}{\sqrt{\in^{2}-\Delta^{2}}}d\in$$

where we used the symmetries $\in_{\xi }=\in_{-\xi }$ and $n_{\xi }\left(T\right)=n_{-\xi }\left(T\right)$. At T=0, Eq. (6) is particularly simple to solve, since $n_{\xi }\left(T=0\right)=0$ for any ξ, and one obtains

(7) $$\frac{1}{\sigma_{0}V}=\text{ln}\left[\sqrt{{\left(\frac{\hbar \omega_{c}}{\Delta }\right)}^{2}+\frac{\hbar \omega_{c}}{\Delta }}\right],$$

which leads to $\sigma_{0}V\ll 1$ [4].

At finite temperatures, the right hand side of Eq. (6) is a monotonically decreasing function of Δ (for any given T below the critical temperature $T_{c}$), so Eq. (6) admits a single solution. At $T=T_{c}$, the solution is Δ=0 and this leads to $k_{B}T_{c}=A\hbar \omega_{c}\text{exp}\left[-1/(\sigma_{0}V)\right]$, where $A=2e^{\gamma }/\pi$ and γ≈0.577 is Euler's constant [4].

### The new set of equations

In Refs. [1], [2], [3] a new procedure was proposed, which eventually imposes some consistency restriction over the standard formalism. The Hamiltonian ${\hat{H}}_{M}$ may be constructed using any parameter μ–not necessary the chemical potential–and then it can be diagonalized, to bring it in the form (2). Denoting the chemical potential by $\mu_{R}$, we can write the partition function as

(8) $$\text{In}\left(Z_{\beta \mu }\right)\equiv -\sum_{ki}\left[\left(1-n_{ki}\right)\text{In}\;(1-n_{ki})+n_{ki}\;\text{In}\;n_{ki}\right]-\beta \left(E-\mu_{R}N\right),$$

which, after the maximization, leads to the quasiparticle populations [1]

(9a) $$n_{ki}\equiv \langle \gamma_{ki}^{†}\gamma_{ki}\rangle =\frac{1}{e^{\beta \left(\in_{k}-{\tilde{\mu }}_{k}\right)}+1},\;i=0,1,$$

where

(9b) $${\tilde{\mu }}_{k}\equiv \frac{\mu_{R}-\mu }{\in_{k}}\left[\xi_{k}-\frac{\sum_{k}\left(1-n_{k0}-n_{k1}\right)\xi_{k}\in_{k}^{-3}}{\sum_{k}\left(1-n_{k0}-n_{k1}\right)\in_{k}^{-3}}\right]$$

is a correction to the quasiparticle energy (in the previous papers we denoted it by $\tilde{\mu }$, omitting the subscript k). The energy gap and the quasiparticle populations are obtained by solving the coupled Eqs. (4) and (9) [1,2]. In the quasi-continuous limit, we assume constant density of states $\sigma_{0}$. Then, Eqs. (4) and (9) may be written as [1,^2^,3]

(10a) $$\frac{2}{\sigma_{0}V}=\int_{-\hbar \omega_{c}}^{\hbar \omega_{c}}\frac{1-2n_{\xi }}{\sqrt{\xi^{2}+\Delta^{2}}}d\xi ,$$

(10b) $$F\left(\mu_{R}-\mu ,T\right)\equiv \frac{\int_{-h\omega_{c}}^{h\omega_{c}}\left(1-2n_{\xi }\right)\frac{\xi }{\in^{3}}d\xi }{\int_{-h\omega_{c}}^{h\omega_{c}}\frac{\left(1-2n_{\xi }\right)d\xi }{\in^{3}}}$$

(10c) $$n_{\xi }\left(\mu_{R}-\mu ,T\right)=\frac{1}{e^{\beta \left[\in_{\xi }-\left(\mu_{R}-\mu \right)\left(\xi -F\right)/\in_{\xi }\right]}+1}.$$

Therefore, instead of Eq. (6) (with the populations given by Eq. 5), that we solve in the standard formalism, we have to solve the system (10), which is more difficult. In order to do that, we write Eq. (10) in dimensionless quantities, defining $x_{F}\equiv \beta F$, x=β∈, y≡βΔ, and $y_{R}\equiv \beta \left(\mu_{R}-\mu \right)$,

(11a) $$x_{F}=\frac{\int_{y}^{\beta \hbar \omega_{c}}\frac{\left(n_{-x}-n_{x}\right)\;dx}{x^{2}}}{\int_{y}^{\beta \hbar \omega_{c}}\frac{\left(1-n_{-x}-n_{x}\right)\;dx}{x^{2}\sqrt{x^{2}-y^{2}}}}\equiv \chi_{F}\left(x_{F},y,y_{R}\right)$$

(11b) $$n_{x}=\frac{1}{e^{x-y_{R}\left(\sqrt{x^{2}-y^{2}}-x_{F}\right)\;/\;x}+1}$$

(11c) $$n_{-x}=\frac{1}{e^{x-y_{R}\left(-\sqrt{x^{2}-y^{2}}-x_{F}\right)\;/\;x}+1}$$

(11d) $$\frac{1}{\sigma_{0}V}=\int_{y}^{\beta \hbar \omega_{c}}\frac{1-n_{-x}-n_{x}}{\sqrt{x^{2}-y^{2}}}dx\equiv I_{\Delta }\left(x_{F},y,y_{R}\right)$$

and introduced the notations $\chi_{F}\left(x_{F},y,y_{R}\right)$ and $I_{\Delta }\left(x_{F},y,y_{R}\right)$ for the expressions in the middle of Eqs. (11a) and (11d). We observe that $n_{\xi }$ and $n_{-\xi }$ (or, equivalently, $n_{x}$ and $n_{-x}$) may be different, so we wrote them explicitly.

Eq. (11) are symmetric under the exchange $y_{R}\to -y_{R}$, $x_{F}\to -x_{F}$, and ξ→−ξ.

#### Low temperature limit

The system of Eq. (11) has multiple solutions and the integrals that appear in Eqs. (11a) and (11d) make it difficult to consistently search for them. For this reason, we start with the study of the equations in the low temperature limit, where we can analytically calculate the integrals and find the solutions easier. As in Ref. [2], we write

(12) $$n_{x}\equiv n_{\xi_{x}}\equiv {\left[\exp\left(\beta m_{\xi_{x}}\right)+1\right]}^{-1}\;\text{and}\;n_{-x}\equiv n_{-\xi_{x}}\equiv {\left[\exp\left(\beta m_{-\xi_{x}}\right)+1\right]}^{-1}$$

where $m_{\xi_{x}}\equiv \left(\Delta /r\right)\left(r^{2}-a\sqrt{r^{2}-1}+ab\right)\equiv m_{\xi }$, $m_{-\xi_{x}}\equiv \left(\Delta /r\right)\left(r^{2}+a\sqrt{r^{2}-1}+ab\right)\equiv m_{-\xi }$, r=∈/Δ=x/y≥1, $a=\left(\mu_{R}-\mu \right)/\Delta =y_{R}/y$, and $b=F/\Delta =x_{F}/y$. The advantage of using the forms (12) is that when β→∞ (i.e., when T→0), $n_{\xi_{x}}$ and $n_{-\xi_{x}}$ converge to either 0 or 1, depending on the signs of $m_{\xi_{x}}$ and $m_{-\xi_{x}}$.

##### The method for finding the quasiparticle population at zero temperature

By defining $t\equiv \xi /\Delta =\pm \sqrt{r^{2}-1}$, we may write $m_{\xi }$ and $m_{-\xi }$ in a single, more convenient form,

(13) $$m_{t}\equiv m_{\xi \left(t\right)}=\frac{\Delta }{\sqrt{t^{2}+1}}\left(t^{2}-at+ab+1\right)\equiv \frac{\Delta }{\sqrt{t^{2}+1}}E\left(t;a,b\right),$$

where E(t;a,b) is a function of t, which depends on the parameters a and b. The sign of E determines the sign of $m_{t}$ and the discriminant of this second order polynomial is $D\equiv a^{2}-4ab-4$. If D>0, then there is an interval $\left(t_{1},t_{2}\right)$, with

(14) $$t_{1,2}=\left(a\mp \sqrt{D}\right)/2,$$

such that, if $t\in (t_{1},t_{2})$, then m(t)<0 and $n_{t}=1$, if $t\notin [t_{1},t_{2}]$, then m(t)>0 and $n_{t}=0$, plus $n_{t_{1}}=n_{t_{2}}=1/2$. The minimum value of E is $E_{min}\left(a,b\right)\equiv E\left(t_{min}=a/2;a,b\right)=-a^{2}/4+ab+1$.

In the following we shall discuss only the case a>0 and the situation a<0 may be obtained from this one, by changing b→−b and t→−t. Then, if D>0, the solutions (20) exist and $t_{2}>0$, whereas $t_{1}>0$ if and only if ab+1>0 ($t_{1}<0$ if ab+1<0). Using these results, we can calculate the quantities in Eq. (11):

(15a) $$\frac{2}{\sigma_{0}V}=\int_{-A}^{A}\frac{dt}{\sqrt{t^{2}+1}}-2\int_{t_{1}}^{t_{2}}\frac{dt}{\sqrt{t^{2}+1}}=2\;\text{In}\left[\sqrt{A^{2}+1}+A\right]-2\left[\arcsin h(t_{2})-\arcsin h(t_{1})\right]$$

(15b) $$b=\frac{\int_{-A}^{A}\frac{\left(1-2n_{t}\right)t}{{\left(t^{2}+1\right)}^{3/2}}dt}{\int_{-A}^{A}\frac{\left(1-2n_{t}\right)}{{\left(t^{2}+1\right)}^{3/2}}dt}=\frac{\int_{-A}^{A}\frac{t\;dt}{{\left(t^{2}+1\right)}^{3/2}}-2\int_{t_{1}}^{t_{2}}\frac{t\;dt}{{\left(t^{2}+1\right)}^{3/2}}}{\int_{-\frac{\hbar \omega_{c}}{\Delta }}^{\frac{\hbar \omega_{c}}{\Delta }}\frac{dt}{{\left(t^{2}+1\right)}^{3/2}}-2\int_{t_{1}}^{t_{2}}\frac{dt}{{\left(t^{2}+1\right)}^{3/2}}}=\frac{\frac{1}{\sqrt{t_{2}^{2}+1}}-\frac{1}{\sqrt{t_{1}^{2}+1}}}{\frac{A}{\sqrt{A^{2}+1}}-\frac{t_{2}}{\sqrt{t_{2}^{2}+1}}+\frac{1}{\sqrt{t_{1}^{2}+1}}}\equiv B\left(A,a,b\right),$$

where we introduced the notation $A\equiv \hbar \omega_{c}/\Delta$, which, in general, is much bigger than 1, and B(A,a,b), which is the expression on the right hand side (r.h.s.) From Eq. (15a) we find the minimum value $A_{0}$ of A, given by the standard zero temperature BCS equation

(16) $$\text{ln}\left[\sqrt{A_{0}^{2}+1}+A_{0}\right]=1/\sigma_{0}V.$$

Furthermore, noticing that

(17) $$\text{arcsinh}\left(t_{1,2}\right)=\text{ln}\left(t_{1,2}+\sqrt{t_{1,2}^{2}+1}\right),$$

Eq. (15a) may be written

(18) $$\frac{\sqrt{t_{1}^{2}+1}+t_{1}}{\sqrt{t_{2}^{2}+1}+t_{2}}=\frac{\Delta }{\Delta_{0}}\frac{\sqrt{1+{\left(1/A_{0}\right)}^{2}}+1}{\sqrt{1+{\left(1/A\right)}^{2}}+1}\approx \frac{\Delta }{\Delta_{0}}\left(1+\frac{1+1/A_{0}^{2}-1/A^{2}}{4}\right)$$

We introduce the notation $M\equiv A/\sqrt{A^{2}+1}$ and $M_{0}\equiv A_{0}/\sqrt{A_{0}^{2}+1}$. Since A≫1 in general, then M≲1. If we chose $\sigma_{0}V=0.2$ and $\Delta =2\times \;10^{-4}$ eV (the value of energy gap in an Al superconductor at zero temperature), we obtain $A_{0}\approx 74.2$ and $1-M\leq 1-M_{0}≲10^{-4}\ll 1$. Nevertheless, in the following we shall work with finite (but large) values of A and we will show that in general the relative differences in the physical quantities calculated with M≲1 and M=1 are of the order of 1−M. Therefore, the approximation M=1 is good enough for all the physically relevant cases. This will make (15b) an equation for b(a), which, if plugged into (15a) or (18), determines Δ(a) [2].

To find the solutions of the system (15) we proceed in two steps: in *Step 1* (*S1*) we find the solution of Eq. (15b) and, using this, in *Step 2* (*S2*) we solve Eq. (15a).

**Fig. 1:**
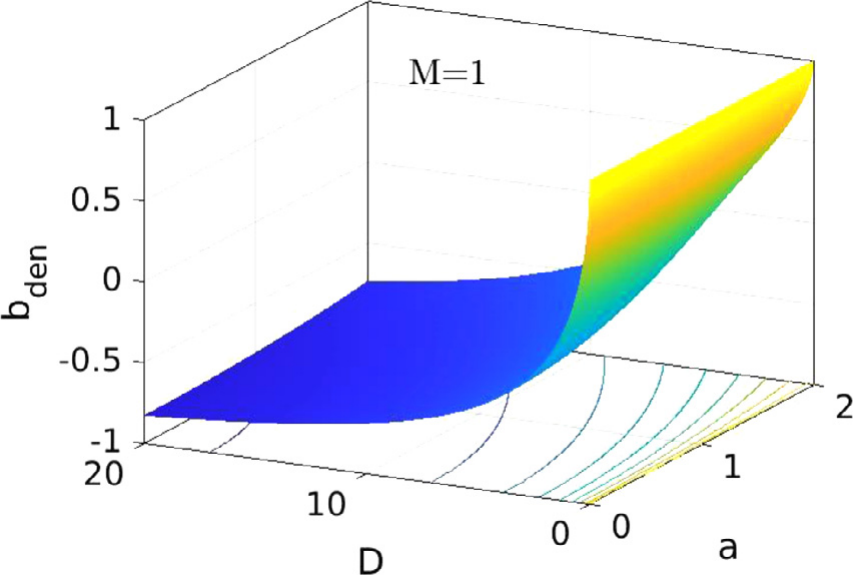
The denominator $B_{\mathrm{den}}$ of the r.h.s. of Eq. (15b), vs. a and D, for M=1 (for realistic values of the parameters, $1-10^{-4}\leq M\leq 1$). The function is monotonically decreasing with D, for fixed a.

*S1* is further divided into smaller sub-steps. In *Step 1.1* (*S1.1*) we take a≤2. In this case, we notice that D≥0 if and only if b≤0, so we loo*k* for negative solutions of Eq. (15b). First, we notice that there is always a solution with b=0, which corresponds to m(t)>0 and $n_{t}=0$ for any t. this solution leads to the typical BCS solutions for Δ at zero temperature. Therefore, we have only to look for the second solution. For this, we notice that the numerator $B_{num}\equiv \frac{1}{\sqrt{t_{2}^{2}+1}}-\frac{1}{\sqrt{t_{1}^{2}+1}}$ of B (Eq. 15b) satisfies $B_{num}\leq 0$, since $t_{2}\geq t_{1}$ (we assume that $B_{num}=0$ and b=0 when D<0), so our solutions are in a range where the denominator $B_{den}\equiv M-\frac{t_{2}}{\sqrt{t_{2}^{2}+1}}+\frac{t_{1}}{\sqrt{t_{1}^{2}+1}}>0$. Using the convention $\frac{t_{2}}{\sqrt{t_{2}^{2}+1}}-\frac{t_{1}}{\sqrt{t_{1}^{2}+1}}\equiv 0$ when D≤0, we obtain $B_{den}\left[D(a,b)\leq 0\right]=M>0$. If we write $t_{1,2}$ as functions of a and D (Eq. 14), then $B_{den}\left(D,a,M\right)$ monotonically decreases with D for any a and M: $B_{den}\left(D=0,a,M\right)=M$ and $B_{den}\left(D\to \infty ,a,M\right)=M-2$, as one can see in Fig. 1. Therefore, the equation $B_{den}\left(D,a,M\right)=0$, as a function of D, has one solution $D_{inv}$ for any M and a. From this, we obtain the value of b where the r.h.s. of Eq. (15b) changes sign, denoted as $b_{inv}\equiv \left(a^{2}-4-D_{inv}\right)/4a$.

In *Step 1.1.1* (*S1.1.1*) we obtain $b_{inv}$ numerically. Practically, this is done by looking for the solution of the equation in b, $B_{den}\left(b,a,M\right)=0$, for fixed a and M, in an interval $b\in (-2/a,b_{0})$, where $b_{0}\equiv \left(a^{2}-4\right)/4a$ is the value at which D=0. We notice that $B_{den}\left(b_{0},a,M\right)=M>0$ and $B_{den}\left(-2/a,a,M\right)<0$ for any a (the second inequality was checked numerically). Then, in *Step 1.1.2* (*S1.1.2*) we find the solution of Eq. (15b) in the interval $\left(b_{0},b_{inv}\right)$

*Step 1.2* (*S1.2*) corresponds to a>2. In this case, $b_{0}\equiv \left(a^{2}-4\right)/4a>0$, so, in principle, a solution b>0 of Eq. (15b) may exist. But, in such a case, the solution $b_{sol}$ should be smaller than $b_{inv}$ [so that $B(A,a,b_{sol})>0$], which implies $b_{inv}\geq 0$. Assuming that $b_{inv}=0$, then

(19) $$M=\frac{\frac{a}{2}+\frac{\sqrt{a^{2}-4}}{2}}{\sqrt{{\left(\frac{a}{2}+\frac{\sqrt{a^{2}-4}}{2}\right)}^{2}+1}}-\frac{\frac{a}{2}-\frac{\sqrt{a^{2}-4}}{2}}{\sqrt{{\left(\frac{a}{2}-\frac{\sqrt{a^{2}-4}}{2}\right)}^{2}+1}}\equiv f_{M}\left(a\right).$$

First, we notice that

(20a) $$f_{M^{\prime }}\left(a\right)=\frac{\sqrt{a+\sqrt{a^{2}-4}}+\sqrt{a-\sqrt{a^{2}-4}}}{\sqrt{2a^{3}\left(a^{2}-4\right)}}>0$$

for any a>2 and

(20b) $$\underset{a\to \infty }{\text{lim}}f_{M}\left(a\right)=1.$$

Then, we calculate the series expansion

(21) $$f_{M}\left(a\right)=1-\frac{1}{a}-\frac{1}{2a^{2}}-O\left(\frac{1}{a^{3}}\right)$$

to obtain (from 21 and 19) that $b_{inv}=0$ if 1/a≈1−M≪1. If M=1, then 1/a=0, so there is no positive solution of Eq. (15b) for any finite a>2. If we denote by $a_{inv}$ the solution of Eq. (19), then, from Eq. (21) we observe that

(22) $$\frac{1}{a_{inv}}+\frac{1}{2a_{inv}^{2}}\approx 1-M,$$

which implies $1/a_{\mathrm{inv}}≲1-M$.

In the general case, when M≲1, if $a\geq a_{inv}$, then $b_{inv}\geq 0$ and, at b=0, we obtain

(23) $$\begin{array}{c} B\left(A,a>a_{\mathrm{inv}},0\right)={\left[1-\frac{\sqrt{2}}{\sqrt{A^{2}+1}}{\left(\sqrt{1+\sqrt{1-\frac{4}{a^{2}}}}-\sqrt{1-\sqrt{1-\frac{4}{a^{2}}}}\right)}^{-1}\right]}^{-1}\;\approx {\left(1-\frac{1}{\sqrt{A^{2}+1}}\right)}^{-1}\\ \left[1+\frac{1}{a\left(\sqrt{A^{2}+1}-1\right)}+\frac{1}{a^{2}\left(\sqrt{A^{2}+1}-1\right)}\left(\frac{3}{2}+\frac{1}{\sqrt{A^{2}+1}-1}\right)\right]≳1 \end{array}$$

(since a and A≫1). To calculate $b_{inv}$, we use the fact that $a>a_{inv}\gg 1$ and $b_{inv}\geq 0$ Using also the assumption that $b_{inv}/a\ll 1$, we obtain the expansion

(24) $$M=\frac{t_{2}}{\sqrt{t_{2}^{2}+1}}-\frac{t_{1}}{\sqrt{t_{1}^{2}+1}}\approx 1-\frac{b_{inv}}{\sqrt{{b_{inv}}^{2}+1}}-\frac{1}{\sqrt{{b_{inv}}^{2}+1}}\frac{1}{a}.$$

Neglecting the term proportional to 1/a, we obtain

(25) $$\frac{1}{b_{inv}^{2}}\approx \frac{1}{{\left(1-M\right)}^{2}}-1<\frac{1}{{\left(1-M\right)}^{2}},$$

which implies $b_{\mathrm{inv}}≳1-M$ and, therefore, $0<b_{inv}\ll 1$. Calculating the derivative, we obtain

(26) $$\frac{\partial B\left(A,a,b\right)}{\partial b}=\frac{1}{B_{den}^{2}\left(A,a,b\right){\left(b^{2}+1\right)}^{3/2}}\left[Mb+\sqrt{b^{2}+1}-\frac{2-M}{a}\right],$$

which is positive, at least for b of the order of $b_{inv}$ (25), and diverges at $b=b_{inv}$. Then, according to (23), (25), and (26), Eq. (15b) does not have any positive solution for a>2 and therefore it does not have any positive solution for any a, at least for the values of M used in this paper, that is, $0<1-M≲10^{-4}\left(\ll 1\right)$.

**Fig. 2:**
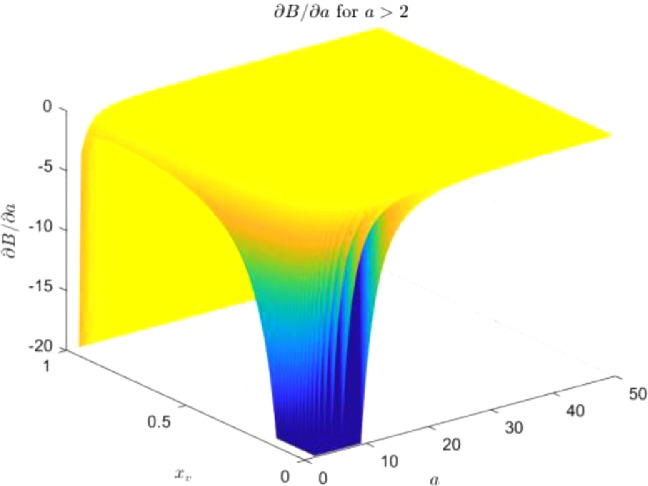
∂B(A,a,b)/∂a vs. a (for a>2) and $x_{v}=b/b_{\mathrm{inv}}\left(a\right)$, for A=74. We observe that it is negative for all the values calculated. When $x_{v}\to 1$, then $b\to b_{\mathrm{inv}}$, and both, $B(A,a,b\to b_{\mathrm{inv}})$ and $\partial B(A,a,b\to b_{\mathrm{inv}})/\partial a$ diverge. The results differ insignificantly from the case A→∞ (i.e., M→1)

To prove that there are also no negative solutions of Eq. (15b) for a>2, we calculate the derivative ∂B(A,a,b)/∂a and numerically show that it is negative (see Fig. 2). Since we can also notice that b≥B(A,a=2,b) for any b<0 [2], we conclude that b≥B(A,a,b) for any b<0 and a>2, so Eq. (15b) has no solution for a>2.

**Fig. 3:**
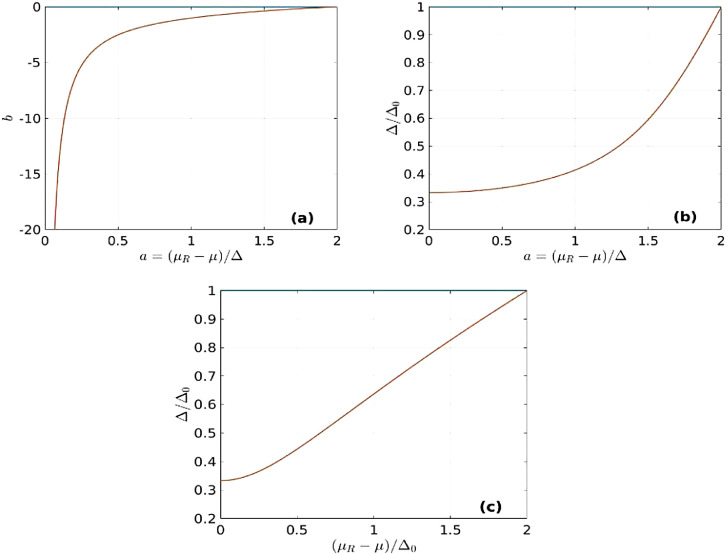
The solution b(a) of Eq. (15b) (a) and the corresponding values of $\Delta /\Delta_{0}$, as functions of $a\equiv (\mu_{R}-\mu )/\Delta$ (b) or $\left(\mu_{R}-\mu \right)/\Delta_{0}$. The blue, horizontal lines in all panels (at b=0 and $\Delta =\Delta_{0}$) correspond to the typical BCS solutions, whereas the red curves represent our second solution. For these plots we used M=1.

Physical quantities at zero temperature and special cases Once we determine the solution b of Eq. (15b), we can proceed to *Step 2* and calculate Δ directly from Eq. (18). Noticing that the relative error in the determination of $\Delta /\Delta_{0}$ is of the order $10^{-4}$ or smaller, we can ignore the effect of finite A and $A_{0}$ and calculate Δ for M=1 (i.e., $A,A_{0}\to \infty$). We shall analyze the situation when a∈[0,2], since for a>2 there are no solutions, whereas for a<0 the solutions are similar to the ones for a>0. As mentioned above, for any a∈[0,2] there are two solutions, the first of which corresponds to b=0 and $\Delta =\Delta_{0}$ (the typical BCS solution at zero temperature), whereas the second solution corresponds to b<0 and $\Delta <\Delta_{0}$, as presented in Fig. 3. We observe that $\text{li}m_{a\to 0}b\left(a\right)=-\infty$, such that $\text{li}m_{a\to 0}ab\left(a\right)\equiv l_{ab}$ is finite (and negative). To calculate the limit $l_{ab}$, we first observe that

(27) $$\underset{a\to 0}{\text{lim}}t_{1,2}\left(a\right)=\mp \sqrt{-ab-1}$$

Since b(a) diverges when a→0 and $B_{num}$ si always finite, then $B_{den}$ should become zero when a→0 and $ab\to l_{ab}$, namely,

(28) $$M=\underset{a\to 0,ab\to l_{ab}}{\text{lim}}\left(\frac{t_{2}}{\sqrt{t_{2}^{2}+1}}-\frac{t_{1}}{\sqrt{t_{1}^{2}+1}}\right)=2\sqrt{\frac{-l_{ab}-1}{-l_{ab}}}$$

which, for M=1, gives [2]

(29) $$l_{ab}=-\frac{4}{3},\;\underset{a\to 0}{\text{lim}}t_{1,2}=\mp \frac{1}{\sqrt{3}},\;\underset{a\to 0}{\text{lim}}\Delta_{2}=\frac{1}{3}$$

where $\Delta_{2}$ is the second solution of the system (15)

**Fig. 4:**
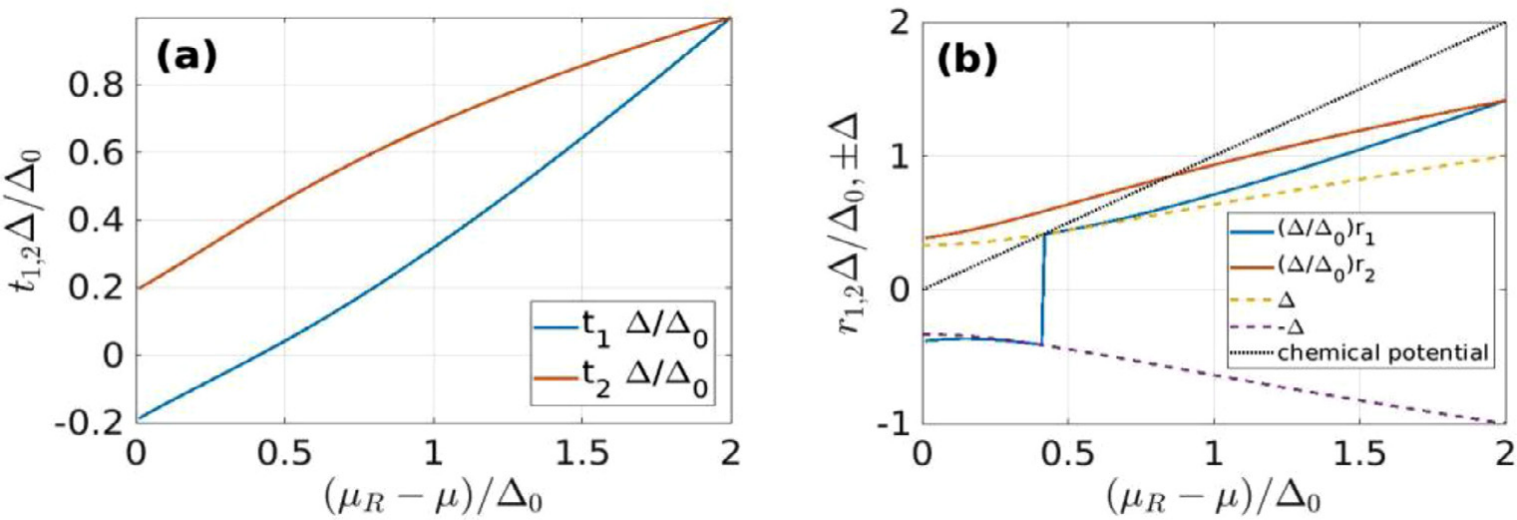
The interval populated by quasiparticles at zero temperature along the free single-particle energy axis $\left[t_{1},t_{2}\right]$ (a) and the BCS quasiparticle axis $\left[r_{1},r_{2}\right]$ (b) vs the scaled asymmetry of the attraction band. In (b) we also show the energy gap [−Δ,Δ] and the relative chemical potential $\left(\mu_{R}-\mu \right)/\Delta_{0}$. At $\mu_{R}-\mu =0$ we have $\Delta /\Delta_{0}=1/3$, $\left(\Delta /\Delta_{0}\right)t_{1,2}=\mp 1/\left(3\sqrt{3}\right)$, $\left(\Delta /\Delta_{0}\right)r_{1,2}=\mathrm{sgn}\left(t_{1,2}\right)2/\left(3\sqrt{3}\right)$.

In Fig. 4 we show the variation of $t_{1,2}$ and $r_{1,2}$ with the scaled asymmetry $\left(\mu_{R}-\mu \right)/\Delta_{0}$. The quantities $r_{1,2}$ represent the scaled BCS quasiparticle energies corresponding to $t_{1,2}$, namely

(30) $$r_{2}=\sqrt{t_{2}^{2}+1}\;\text{and}\;r_{1}=sgn\left(t_{1}\right)\sqrt{t_{1}^{2}+1}$$

In (b) we also plot the energy gap (±Δ) and the relative chemical potential $(\mu_{R}-\mu )/\Delta$, for exemplification.

##### Conservation of the number of particles

**Fig. 5:**
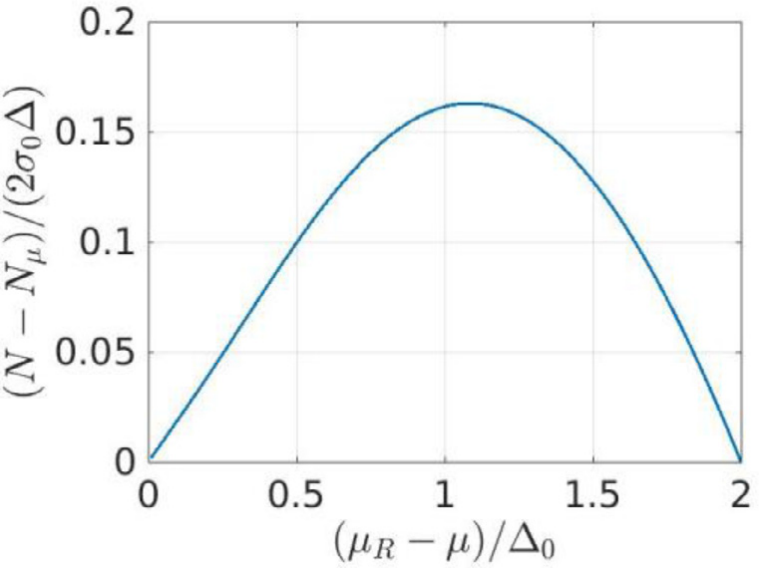
The variation of the total number of particles N with the asymmetry of the attraction band.

For the first solution of Eq. (15), the average total number of particle N is equal to $N_{\mu }$, which is the number of states up to the free single-particle energy μ. For the second solution, the total number of particles in the low temperature limit is [1,2]

(31) $$\langle N\rangle \equiv N=N_{\mu }+2\sigma_{0}\int_{-\hbar \omega_{c}}^{\hbar \omega_{c}}\frac{\xi n_{\xi }}{\in }d\xi =N_{\mu }+2\sigma_{0}\Delta \left(\sqrt{t_{2}^{2}+1}-\sqrt{t_{1}^{2}+1}\right),$$

where we used the notation 〈N〉 to show explicitly that the total number of particles N represents an average value. In Fig. 5 we plot the function $\left(N-N_{\mu }\right)/\left(2\sigma_{0}\Delta \right)$. Therefore, in the case when N is fixed and is different from $N_{\mu }$, only the second solution may be realized and $\mu_{R}$ becomes a function of μ.

### Finite temperatures

To find the solutions of Eq. (15), we start from the solutions at T=0, obtained in the Section 1.2.1. In this case, analytical calculations are more difficult, so our investigations are mostly numerical. The function $\chi_{F}\left(x_{F},y,Y_{R}\right)$ (11a) is the finite temperature correspondent of the function B(A,a,b) (15b) used in Section 1.2.1. As we did there, we define

(32) $$\chi_{F,num}\left(x_{F},y,y_{R}\right)\equiv \int_{y}^{\beta \hbar \omega_{c}}\frac{\left(n_{-x}-n_{x}\right)dx}{x^{2}}\;and\;\chi_{F,den}\left(x_{F},y,y_{R}\right)\equiv \int_{y}^{\beta \hbar \omega_{c}}\frac{\left(1-n_{-x}-n_{x}\right)dx}{x^{2}\sqrt{x^{2}-y^{2}}}.$$

**Fig. 6:**
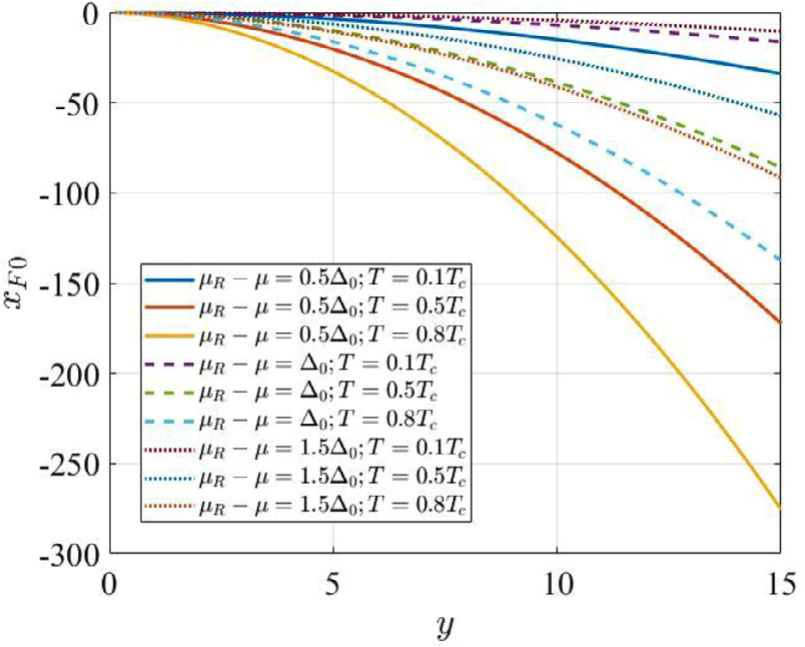
The solutions $x_{F,0}\left[y,\left(\mu_{R}-\mu \right)/\left(k_{B}T\right)\right]$ of the equation $\chi_{F,den}\left[x_{F},y,\left(\mu_{R}-\mu \right)/\left(k_{B}T\right)\right]=0$ in $x_{F}$, for different values of $\mu_{R}-\mu$ and $k_{B}T$.

**Fig. 7:**
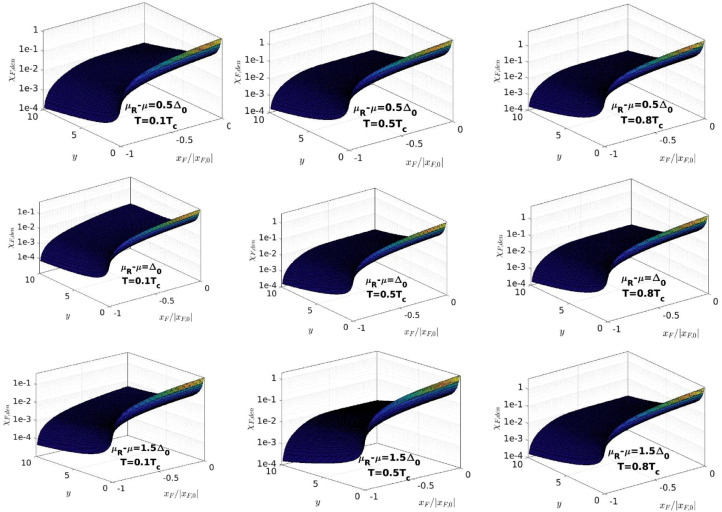
The dependence of $\chi_{F,den}$ on $x_{F}/\left|x_{F,0}\right|$ and y for the values of $\mu_{R}-\mu$ and T shown in each panel. The values of $x_{F,0}$ are plotted in Fig. 6. We use the logarithmic scale on the vertical axis to observe the variation of $\chi_{F,den}$ with $x_{F}$ for any value of y. For $x_{F}\geq x_{F,0}$, $\chi_{F,den}$ is negative, so we do not plot the function in this region.

We notice that for positive $y_{R}$, $\chi_{F,num}\left(x_{F},y,y_{R}\right)$ is negative, whereas $\chi_{F,den}\left(x_{F},y,y_{R}\right)$ changes sign as a function of $x_{F}$, going through zero at some point $x_{F,0}\left(y,y_{R}\right)$. Being a monotonically increasing function of $x_{F}$ (see Fig. 7, for the positive part of $\chi_{F,den}\left(x_{F},y,y_{R}\right)$), the zero of $\chi_{F,den}\left(x_{F},y,y_{R}\right)$ is easily found numerically for any fixed y and $y_{R}$. In Fig. 6 we plot $x_{F,0}\left(y,y_{R}\right)$ for some relevant values of the parameters $\mu_{R}-\mu$ and T ($y_{R}=\frac{\mu_{R}-\mu }{k_{B}T}\equiv \frac{\Delta_{0}}{k_{B}T_{c}}\frac{\mu_{R}-\mu }{\Delta_{0}}\frac{T_{c}}{T}$, where $\Delta_{0}/\left(k_{B}T_{c}\right)=2/A$ and A was defined aboves [4]). In Fig. 7 we exemplify the dependence of $\chi_{F,den}\left(x_{F},y,y_{R}\right)$ on $x_{F}/x_{F,0}$ and y, for the values of $\mu_{R}-\mu$ and T used in Fig. 6–we use the variable $x_{F}/x_{F,0}$ in order to re-scale the intervals $\left(x_{F,0}\left(y,y_{R}\right),0\right)$ into the interval (0,1) for all the values of y and $y_{R}$. For $x_{F}<x_{F,0}$ the function is negative.

**Fig. 8:**
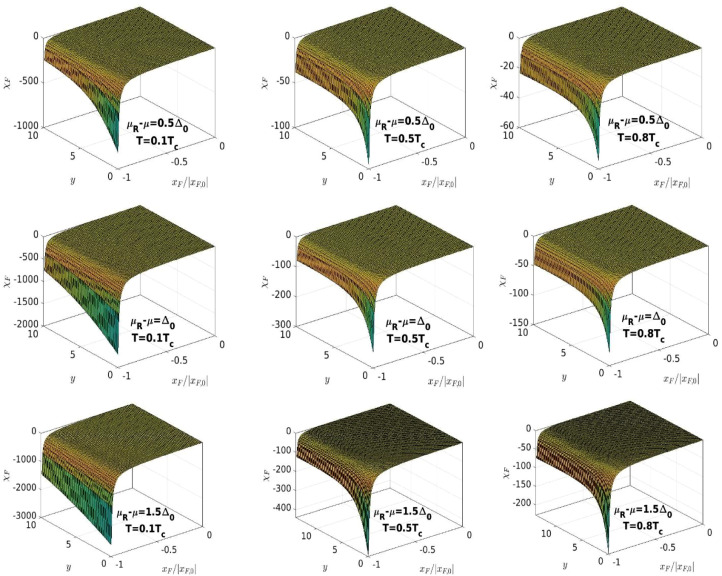
The dependence of $\chi_{F}$ on $x_{F}/\left|x_{F,0}\right|$ and y for the values of $\mu_{R}-\mu$ and T shown in each panel. The values of $x_{F,0}$ are plotted in Fig. 6.

At $x_{F}=x_{F,0}\left(y,y_{R}\right)$, $\chi_{F}\left(x_{F},y,y_{R}\right)$ is divergent. Since $\chi_{F}\left(x_{F},y,y_{R}\right)$ is also monotonically decreasing with $|x_{F}|$, Eq. (11a) has a single solution in the interval $\left(x_{F,0}\left(y,y_{R}\right),0\right)$, which can be found using a simple numerical algorithm. In Fig. 8 we plot $\chi_{F}$ as a function of $x_{F}/\left|x_{F,0}\right|$ and y (as we did in Fig. 7), for the values of $\mu_{R}-\mu$ and T used in Figs. 6 and 7, to emphasize the general behavior of the function, especially its divergence at $x_{F}=x_{F,0}\left(y,y_{R}\right)$.

**Fig. 9:**
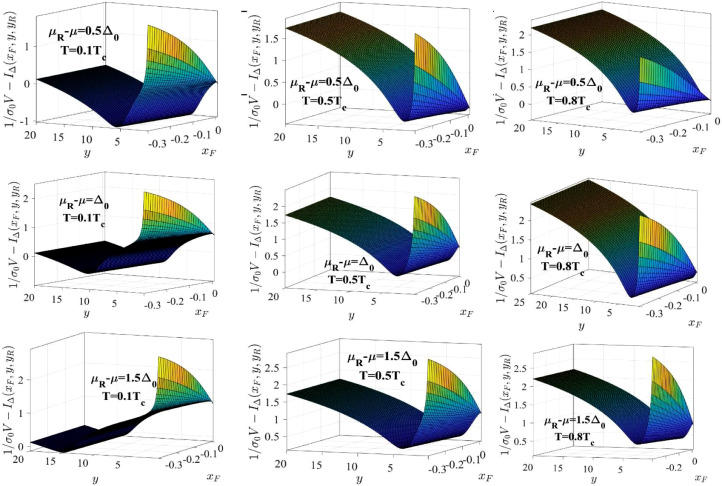
The dependence of $1/\left(\sigma_{0}V\right)-I_{\Delta }\left(x_{F},y,y_{R}\right)$ on $x_{F}/\left|x_{F,0}\right|$ and y for the values of $\mu_{R}-\mu$ and T shown in each panel. The values of $x_{F,0}$ are plotted in Fig. 6.

Once the solution of Eq. (11a) is obtained, which is a function $x_{F}\left(y,y_{R}\right)$, we can plug it into Eq. (11d) and solve for y, for given $y_{R}$. In Fig. 9 we plot $1/\left(\sigma_{0}V\right)-I_{\Delta }\left(x_{F},y,y_{R}\right)$ and we see that the function may have two solutions in y for some values of $x_{F}$ and $y_{R}$. Because of this property, Eq. (11d), with $x_{F}$ replaced by the function $x_{F}\left(y,y_{R}\right)$, obtained from Eq. (11a), has two solutions, as shown in the accompanying paper [3].
